# Carotid body responses to O_2_ and CO_2_ in hypoxia‐tolerant naked mole rats

**DOI:** 10.1111/apha.13851

**Published:** 2022-06-27

**Authors:** Ying‐Jie Peng, Jayasri Nanduri, Ning Wang, Shakil A. Khan, Matthew E. Pamenter, Nanduri R. Prabhakar

**Affiliations:** ^1^ Institute for Integrative Physiology and Center for Systems Biology of O_2_ Sensing University of Chicago Chicago Illinois USA; ^2^ Department of Biology University of Ottawa Ottawa Ontario Canada; ^3^ University of Ottawa Brain and Mind Research Institute Ottawa Ontario Canada

**Keywords:** carbon monoxide, carbonic anhydrase, heme oxygenase, hydrogen sulfide, hypercapnia, hypercapnic ventilatory response, hypoxia, hypoxic ventilatory response

## Abstract

**Aim:**

Naked mole rats (NMRs) exhibit blunted hypoxic (HVR) and hypercapnic ventilatory responses (HCVR). The mechanism(s) underlying these responses are largely unknown. We hypothesized that attenuated carotid body (CB) sensitivity to hypoxia and hypercapnia contributes to the near absence of ventilatory responses to hypoxia and CO_2_ in NMRs.

**Methods:**

We measured ex vivo CB sensory nerve activity, phrenic nerve activity (an estimation of ventilation), and blood gases in urethane‐anesthetized NMRs and C57BL/6 mice breathing normoxic, hypoxic, or hypercapnic gases. CB morphology, carbon monoxide, and H_2_S levels were also determined.

**Results:**

Relative to mice, NMRs had blunted CB and HVR. Morphologically, NMRs have larger CBs, which contained more glomus cells than in mice. Furthermore, NMR glomus cells form a dispersed pattern compared to a clustered pattern in mice. Hemeoxygenase (HO)‐1 mRNA was elevated in NMR CBs, and an HO inhibitor increased CB sensitivity to hypoxia in NMRs. This increase was blocked by an H_2_S synthesis inhibitor, suggesting that interrupted gas messenger signaling contributes to the blunted CB responses and HVR in NMRs. Regarding hypercapnia, CB and ventilatory responses to CO_2_ in NMRs were larger than in mice. Carbonic anhydrase (CA)‐2 mRNA is elevated in NMR CBs, and a CA inhibitor blocked the augmented CB response to CO_2_ in NMRs, indicating CA activity regulates augmented CB response to CO_2_.

**Conclusions:**

Consistent with our hypothesis, impaired CB responses to hypoxia contribute in part to the blunted HVR in NMRs. Conversely, the HCVR and CB are more sensitive to CO_2_ in NMRs.

## 1 INTRODUCTION

Naked mole rats (NMRs; *Heterocephalus glaber*) are subterranean and eusocial rodents, who reside in crowded underground burrows.^1^, ^2^ A notable feature of NMRs is their remarkable tolerance to hypoxia, as they can survive for hours and weeks at 3% and 8% O_2_, respectively,^3^, ^4^ and up to 18 min in an anoxic environment.^5^ NMRs are also highly tolerant to environmental hypercapnia. For example, NMRs survive several hours breathing 80% CO_2_, which is lethal to mice within minutes.^5^ Moreover, NMRs do not exhibit metabolic, thermoregulatory, or behavioral changes to hypercapnia (<10% CO_2_).^6^, ^7^ Conversely, acute hypoxia suppresses the metabolic rate of NMRs,^4^ which is in part due to modest reductions in physical activity and a total cessation of thermoregulation, resulting in decreased body temperature to near ambient levels in hypoxia.^8^, ^9^, ^10^

Unlike NMRs, most adult mammals do not produce robust suppression of metabolic rate during hypoxia. Instead, rely more so on the hypoxic ventilatory response (HVR, i.e., a reflex increase in ventilation in hypoxia) to enhance the delivery of O_2_ to tissues when environmental O_2_ is limited.^11^ Similarly, most adult mammals respond to increased ventilation by CO_2_ (hypercapnic ventilatory response or HCVR).^12^ Intriguingly, whereas most mammals respond to hypoxia or hypercapnia with an increase in ventilation,^11^, ^13^ NMRs manifest a blunted HVR (7% inspired O_2_) and also a blunted HCVR.^5^, ^7^ The mechanism(s) underlying the attenuated HVR and HCVR in NMRs are not known.

Carotid bodies (CBs) are the major sensory organs for monitoring the chemical composition of arterial blood, particularly in hypoxemia and to a lesser extent hypercarbia.^14^ The chemoreceptor tissue in CBs is composed of O_2_‐sensitive glomus cells and supporting type II cells.^14^ Hypoxemia stimulates carotid sinus nerve (CSN) activity, triggering a reflex increase in breathing (i.e., the HVR). On the other hand, the CB chemoreflex underlies ~20%–40% of the HCVR in humans, while the remaining 60%–80% is mediated by the central chemoreceptor(s) located in the brainstem.^15^, ^16^, ^17^ Given that the CB plays a major role in mediating the HVR, and to a lesser extent the HCVR, we hypothesized that impaired CB responses to hypoxia and hypercapnia contribute in part to the blunted HVR and HCVR in NMRs. We tested this hypothesis by monitoring CSN and efferent phrenic nerve responses to hypoxia and hypercapnia in urethane‐anesthetized NMRs and C57BL/6 (BL6) mice, which we have previously studied.^5^, ^7^ Two approaches were employed to assess CB function: first by monitoring breathing responses to brief hyperoxia (i.e., the Dejour's test^18^), which is an established assay for determining CB O_2_ sensitivity in humans^18^ and rodents,^18^, ^19^ and second by directly measuring CSN activity in an ex vivo CB preparation. We chose the ex vivo CB preparation to exclude confounding influences from blood pressure changes on CSN activity,^20^ which is commonly encountered in intact anesthetized animal preparations.

## 2 RESULTS

### 2.1 CB responses to hypoxia are impaired in NMRs

#### 2.1.1 Ventilatory responses to brief hyperoxia (Dejour's test)

**FIGURE 1:**
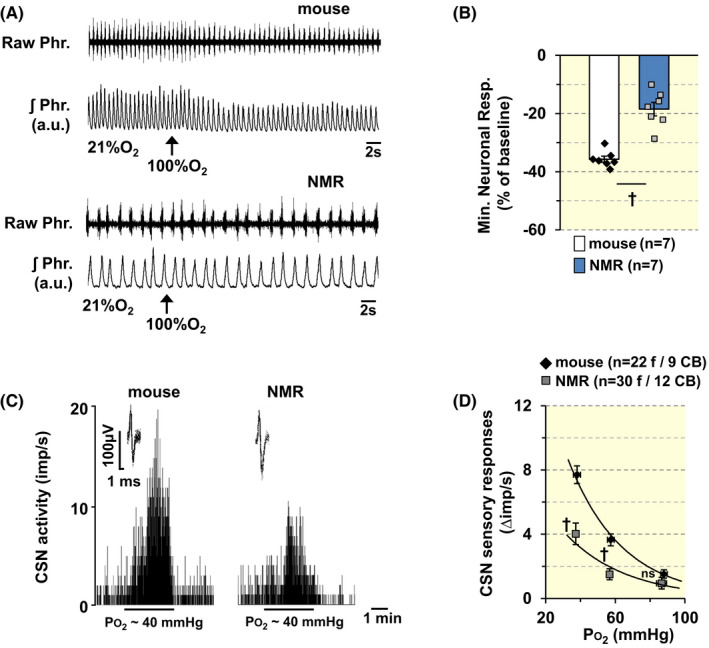
Carotid body (CB) response to hypoxia is impaired in NMRs. (A). Phrenic nerve response to brief hyperoxia (100% O_2_) was determined as an index of CB sensitivity to O_2_ (Dejour's test). Examples of phrenic nerve responses to 30‐s exposures to 100% O_2_ (at arrow) in spontaneously breathing, urethane anesthetized BL6 mouse, and NMR are shown. Raw Phr., action potentials of the phrenic nerve. ʃPhr, integrated phrenic nerve activity. (B). Individual data points along with mean ± SEM are presented as minute neural respiration response to hyperoxia as percent of baseline breathing in room air (control). Numbers in parenthesis represent the number of animals, **p < 0.01, Mann–Whitney test. (C). Examples of CB sensory nerve (CSN) response to hypoxia (medium PO_2_ ~ 40 mmHg) in BL6 mice and NMR. Integrated action potential frequency presented as impulses per second (imp/s). *Insets*: superimposed action potentials of a “single” fiber from which data were derived. (D). Average data (mean ± SEM) of CSN responses to graded hypoxia presented as hypoxia‐evoked CSN response minus baseline CSN activity (Δ impulse/s). Numbers in parenthesis represent the number of fibers and number of CBs (*n* = 5 BL6 mice; 6 NMRs), **p < 0.01; n.s., *p* > 0.05, BL6 vs. NMR (from left to right, *p* < 0.001, *p* < 0.001, and *p* = 0.217, respectively), two‐way ANOVA with repeated measures followed by Holm‐Sidak test. For factor of species, *F*_(1,100)_ = 46.447, *p* < 0.001; for O_2_ level, *F*_(2,100)_ = 124.24, *p* < 0.001; for Species × O_2_ level, *F*_(2,100)_ = 13.116, *p* < 0.001.

Baseline phrenic nerve activity was recorded in urethane‐anesthetized animals breathing room air. Animals were challenged with 100% O_2_ (hyperoxia) for 30 s. Changes in phrenic nerve activity were analyzed during the last 20 s of the hyperoxic challenge. The initial 10s were excluded to account for the dead space in the breathing circuit. Brief hyperoxia depressed breathing in mice, but not in NMRs (Figure 1A,B; *p* < 0.01; *n* = 7 animals for each species).

#### 2.1.2 CSN responses to hypoxia

Examples of CSN responses to hypoxia in a mouse and an NMR are shown in Figure 1C. Overall, CSN responses to hypoxia were blunted in NMRs compared to mice. Specifically, CSN responses to severe (Po_2_ ~ 40 mmHg) and moderate hypoxia (Po_2_ ~ 56 mmHg) were significantly attenuated in NMRs compared to mice (Figure 1D; *p* < 0.01; *n* = 9 CBs from 5 mice and 12 CBs from 6 NMRs).

### 2.2 NMRs have a blunted HVR

The CB chemoreflex is a major driver of the HVR.^14^ To assess whether the blunted CSN response to hypoxia is reflected in the HVR, we measured efferent phrenic nerve responses to a range of inspired O_2_ levels in spontaneously breathing urethane‐anesthetized animals. Body temperature was maintained at either 38 ± 1°C in mice or 33 ± 1°C in NMRs, which are their respective physiological body temperatures.^21^

**FIGURE 2:**
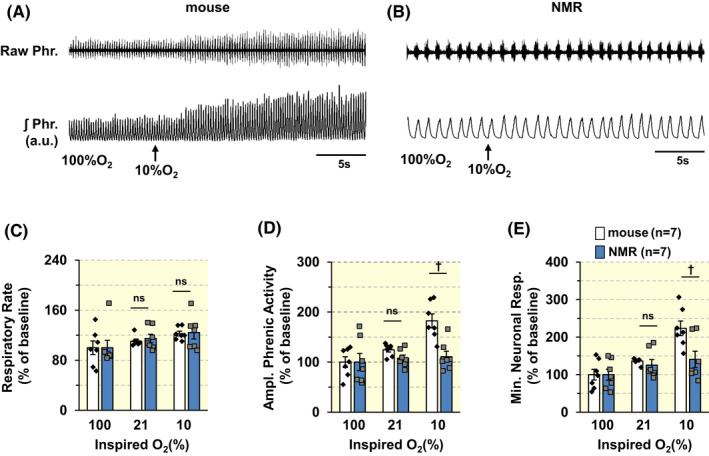
Phrenic nerve responses to hypoxia are attenuated in NMRs. (A, B). Examples of efferent phrenic nerve activity during 100 and 10% inspired O_2_ in urethane anesthetized, spontaneously breathing BL6 mouse (A) and an NMR (B). Raw Phr., phrenic nerve action potentials. ʃPhr., integrated efferent phrenic nerve activity (arbitrary units; a.u.). Arrows indicate administration of hypoxic gas mixture. (C–E). Individual and average (mean ± SEM) data of changes in respiratory rate (RR; phrenic burst frequency/min; (C), tidal amplitude of phrenic activity (D), and minute neuronal respiration (E) of BL6 mice (*n* = 7) and NMRs (*n* = 7) with varying levels inspired O_2_ levels. Data presented as percent of inspiring 100% O_2_. ^†^ *p* < 0.01; n.s., *p* > 0.05, BL6 vs. NMRs, two‐way ANOVA with repeated measures followed by Holm‐Sidak test. In B, for factor of Species, *F*_(1,24)_ = 0.0375, *p* = 0.849; for Oxygen level, *F*_(2,24)_ = 5.649, *p* = 0.01; for Species × Oxygen level, *F*_(2,24)_ = 0.0551, *p* = 0.947. In C, for factor of Species, *F*_(1,24)_ = 12.871, *p* = 0.004; for Oxygen level, *F*_(2,24)_ = 57.757, *p* = 0.03; for Species × Oxygen level, *F*_(2,24)_ = 4.821, *p* = 0.017. In D, for factor of Species, *F*_(1,24)_ = 5.338, *p* = 0.039; for Oxygen level, *F*_(2,24)_ = 14.827, *p* < 0.001; for Species × Oxygen level, *F*_(2,24)_ = 4.357, *p* = 0.024.

Figure 2A,B depict representative examples of breathing responses to 10% O_2_ in a mouse and an NMR. Overall, NMRs had a lower baseline respiratory rate (phrenic burst frequency per min) than mice. Upon exposure to 10% O_2_, phrenic burst frequency and tidal phrenic amplitude increased in mice, whereas these effects were nearly absent in NMRs (Figure 2A,B). Specifically, baseline respiratory rate (RR, phrenic bursts/min) and minute neural respiration (MNR) were significantly less in NMRs compared to mice (Table 1, mice vs. NMR, *p* < 0.01; †). Because the baseline breathing was different between NMRs and mice, we analyzed the HVR as a percent of baseline breathing in animals breathing 100% O_2_ (Figure 2C–E). Compared to mice, NMRs manifested an attenuated HVR in 10% O_2_, which was due to lesser increase in tidal phrenic amplitude (i.e., tidal volume) and minute neural respiration (MNR) than mice (Figure 2D,E; *p* < 0.01, ^†^ *n* = 7 animals for each species).

**TABLE 1:** Phrenic nerve responses to three levels of inspired O_2_ in urethane‐anesthetized NMRs and mice

	100% O_2_	21% O_2_	10% O_2_
*Mice (n = 7)*			
RR (breaths/min)	160 ± 17.3	177 ± 19.5	195 ± 20.0
Tidal Ampl (a.u.)	3.0 ± 0.3	3.8 ± 0.5	5.4 ± 0.7
MNR (a.u.∙min)	475 ± 67.9	644 ± 88.2	1021 ± 118.4
*NMRs (n = 7)*			
RR (breaths/min)	48 ± 5.7^†^	56 ± 10.0	62 ± 13.2
Tidal Ampl (a.u.)	2.9 ± 0.5^ns^	3.0 ± 0.4	3.0 ± 0.4
MNR (a.u.∙min)	133 ± 19.2^†^	159 ± 23.3	177 ± 29.8

Abbreviations: MNR, minute neuronal respiration; RR, respiratory rate; Tidal Ampl, tidal phrenic amplitude (arbitrary units; a.u.).

^†^ *p* < 0.01.

^ns^ *p* > 0.05, mice vs. NMRs, Mann–Whitney test.

Arterial blood gases were also measured in NMRs and mice breathing while breathing either room air or 10% O_2_ gas mixture. NMRs had a lower Pao_2_ than BL6 while breathing either room air (21% O_2_) or hypoxic gas (10% O_2_) (Table 2, BL6 vs. NMR, *p* < 0.05).

**TABLE 2:** Arterial blood gas values with two levels of inspired O_2_ levels in urethane‐anesthetized NMRs and mice

	21% O_2_	10% O_2_
Mice (37°C)	(*n* = 7)	(*n* = 5)
*Pa*_O2_ (mmHg)	96 ± 6	39 ± 2
*Pa*_CO2_ (mmHg)	36 ± 3	33 ± 2
*p*H	7.31 ± 0.04	7.24 ± 0.04
NMRs (33°C)	(*n* = 7)	(*n* = 6)
*Pa*_O2_ (mmHg)	75 ± 7*	31 ± 3*
*Pa*_CO2_ (mmHg)	28 ± 4*	27 ± 3*
*p*H	7.36 ± 0.04^ns^	7.31 ± 0.03^ns^

* *p* < 0.05.

^ns^ *p* > 0.05, mice vs. NMRs, Mann–Whitney test or *t*‐test.

### 2.3 CB and breathing responses to CO_2_ in NMRs

#### 2.3.1 CB response to CO_2_

**FIGURE 3:**
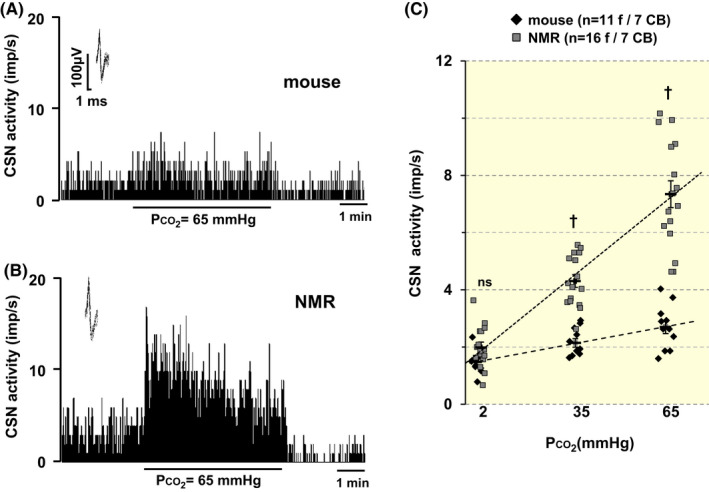
Carotid body responses to CO_2_ are augmented in NMR. (A, B) Examples of carotid body sensory nerve (CSN) responses to hyperoxic hypercapnia (medium PO_2_ ~ 500 mmHg, PCO_2_ ~ 65 mmHg) in a BL6 mouse (A) and an NMR (B). CSN action potential frequency is presented as impulses per second (imp/s). *Insets*: superimposed action potentials of a “single” fiber from which data were derived. (C). Individual and average data (mean ± SEM) of CSN activity (impulse/s). Numbers in parenthesis represent the number of fibers and number of CBs from 6 mice and NMRs each. PCO_2_ = partial pressure of CO_2_ in the medium irrigating the ex vivo CBs. ^†^ *p* < 0.01; n.s., *p* > 0.05, BL6 vs. NMR (from left to right, *p* = 0.218, *p* < 0.001, and *p* < 0.001, respectively), two‐way ANOVA with repeated measures followed by Holm‐Sidak test. For factor of species, *F*_(1,50)_ = 52.689, *p* < 0.001; for CO_2_ level, *F*_(2,50)_ = 70.883, *p* < 0.001; for species × CO_2_ level, *F*_(2,50)_ = 51.78, *p* < 0.001.

CSN responses to hypercapnia were examined in NMRs and mice. Figure 3A,B depict representative examples of CSN responses to CO_2_ in a mouse and an NMR and average data with graded hypercapnia is presented in Figure 3C. Overall, NMRs had greater CSN responses to CO_2_ than BL6 mice (*p* < 0.01; *n* = 7 CBs in each species; 4 animals for each species).

#### 2.3.2 HCVR

**FIGURE 4:**
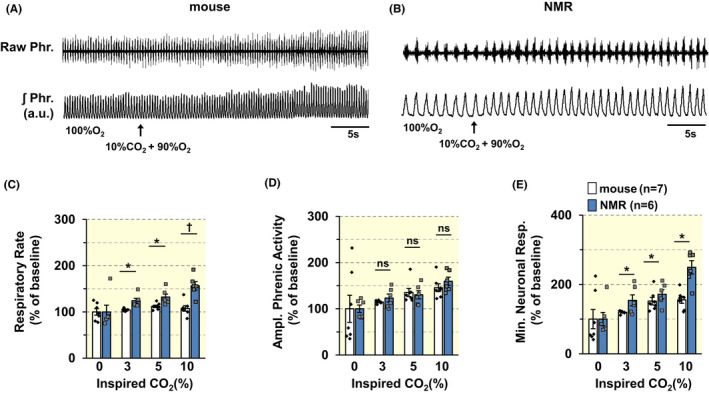
Phrenic nerve responses to hypercapnia are augmented in NMRs. (A, B). Examples of phrenic nerve responses to 10% CO_2_ + 90% O_2_ in urethane anesthetized, spontaneously breathing in a BL6 mouse (A) and an NMR (B). Raw Phr., phrenic nerve action potentials. ʃPhr., integrated efferent phrenic nerve activity (arbitrary units; a.u.). Arrows indicate application of hypercapnia. (C–E). Individual and average data (mean ± SEM) of changes in respiratory rate (RR; phrenic burst frequency/min; (C), tidal amplitude of phrenic activity (D), and minute neuronal respiration (E) in response to graded inspired CO_2_ levels in BL6 mice (*n* = 7) and NMRs (*n* = 6). ^†^ *p* < 0.01; *p < 0.05; n.s., *p* > 0.05, BL6 vs. NMRs, two‐way ANOVA with repeated measures followed by Holm‐Sidak test. In B, for factor of species, *F*_(1,33)_ = 12.487, *p* = 0.005; for CO_2_ level, *F*_(3,33)_ = 8.89, *p* < 0.001; for species × CO_2_ level, *F*_(3,33)_ = 4.127, *p* = 0.014. In C, for factor of species, *F*_(1,33)_ = 0.18, *p* = 0.679; for CO_2_ level, *F*_(3,33)_ = 5.784, *p* = 0.003; for species × CO_2_ level, *F*_(3,33)_ = 0.211, *p* = 0.888. In D, for factor of Species, *F*_(1,33)_ = 8.233, *p* = 0.015; for CO_2_ level, *F*_(3,33)_ = 15.055, *p* < 0.001; for species × CO_2_ level, *F*_(3,33)_ = 3.307, *p* = 0.032.

Representative examples of phrenic nerve responses to 10% CO_2_ in a mouse and an NMR are shown in Figure 4A,B. Average data of absolute values of phrenic nerve responses are presented in Table 3 and HCVR data analyzed as a percent of baseline ventilation while breathing 90% O_2_ is shown in Figure 4C–E. The magnitude of the hypercapnia‐mediated increase in minute neural respiration (MNR) was higher in NMRs than mice, which was due to a greater increase in respiratory rate (i.e., phrenic burst frequency) (Figure 4C,E; mice vs. NMR *p* < 0.01; *n* = 7 mice; 6 NMRs).

**TABLE 3:** Phrenic nerve responses to graded hypercapnia in urethane‐anesthetized NMRs and mice

	0% CO_2_	3% CO_2_	5% CO_2_	10% CO_2_
*Mice (n = 7)*				
RR (breaths/min)	167 ± 11.7	174 ± 11.8	187 ± 14.9	180 ± 15.9
Tidal Ampl (a.u.)	2.9 ± 0.9	3.3 ± 0.9	3.8 ± 1.0	4.1 ± 1.1
MNR (a.u.∙min)	457 ± 127.1	539.2 ± 142.5	660 ± 159.9	684 ± 171.8
*NMRs (n = 6)*				
RR (breaths/min)	51 ± 7.4^**†**^	62 ± 8.2	67 ± 9.6	79 ± 11.1
Tidal Ampl (a.u.)	3.0 ± 0.2^ns^	3.8 ± 0.5	4.0 ± 0.5	4.9 ± 0.7
MNR (a.u. min)	156 ± 30.4*	231 ± 34.3	266 ± 49.3	391 ± 78.3

*Note*: Numbers in parenthesis represent number of animals.

Abbreviations: MNR, minute neuronal respiration; RR, respiratory rate; Tidal Ampl, tidal phrenic amplitude (arbitrary units; a.u.).

* *p* < 0.05.

^†^ *p* < 0.01.

^ns^ *p* > 0.05, mice vs. NMRs, Mann–Whitney test.

### 2.4 CB morphology

**FIGURE 5:**
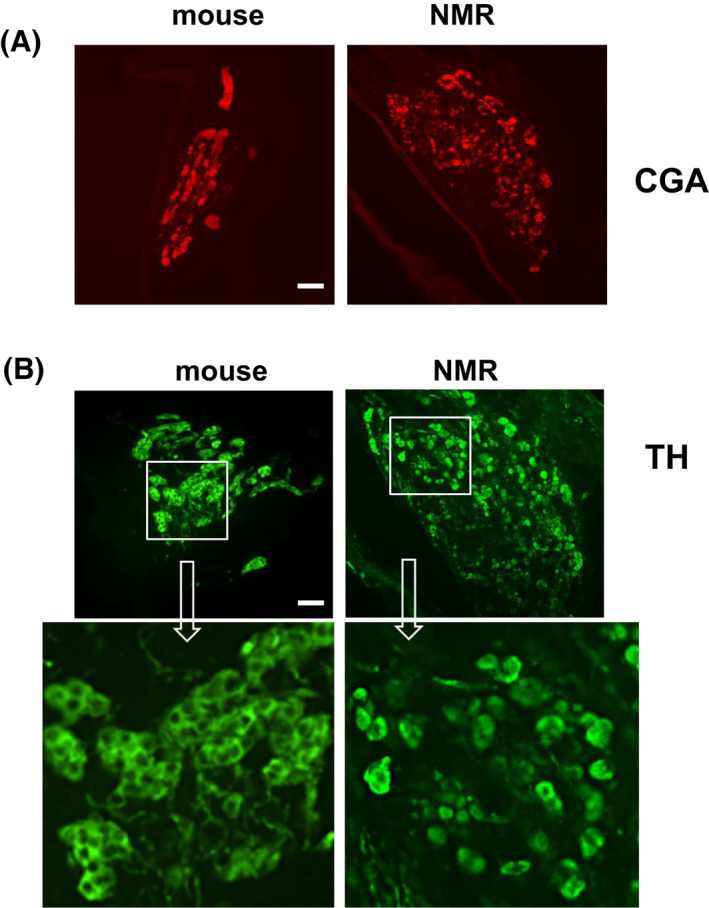
Morphology of the carotid body. (A, B). Examples of carotid body sections stained with either anti chromogranin A (CGA; A) or anti tyrosine hydroxylase (TH) antibodies (B). Upper panel in B represents low magnification image and higher magnification in lower panel. BL6 = CBs sections from BL6 mouse and an NMR. Scale bar = 50 μm. Note the clustered distribution pattern of glomus cells in BL6 CB section and dispersed pattern in NMRs. *N* = 4 BL6 and NMRs each.

To assess CB morphology, CB sections from NMRs and mice were stained with anti‐TH and ant‐CGA antibodies, which are established markers of glomus cells,^14^, ^22^ and morphometric analysis was performed as described in the “Methods” section. CBs were bigger in NMRs than mice, as indicated by a greater CB volume (~230%) in NMRs compared to CBs [Figure 5A; Table 4, mice vs. NMR, *p* < 0.05; *n* = 4 each species]. The number of TH and CGA positive cells was higher in NMR CBs but the ratio of TH or CGA positive cells to the CB volume was comparable between NMRs and BL6 (Table 4; BL6 vs. NMR, *p* > 0.05). NMR glomus cells formed a dispersed pattern compared to a clustered pattern in BL6 mice CBs (Figure 5B).

**TABLE 4:** Morphometric analysis of the carotid body (CB) in mice and NMRs

	CB volume	TH volume	CGA volume	TH/CB	CGA/CB
	(×10^5^ μm^3^)	(×10^4^ μm^3^)	(×10^4^ μm^3^)	(%)	(%)
Mice	2.1 ± 0.14	4.3 ± 0.28	2.8 ± 0.1	20.2 ± 0.3	15.7 ± 0.9
NMRs	4.9 ± 0.7*	9.1 ± 1.1^**†**^	6.8 ± 0.98*	19 ± 0.4^ns^	13.9 ± 0.2^ns^

*Note*: Data were presented as Mean ± SEM. *N* = 4 each species.

Abbreviations: CGA, chromogranin A; TH, tyrosine hydroxylase.

* *p* < 0.05.

^†^ *p* < 0.01.

^ns^ *p* > 0.05, not significant, mice vs. NMRs, Mann–Whitney test.

### 2.5 CO‐H_2_S signaling is interrupted in NMR CBs

**FIGURE 6:**
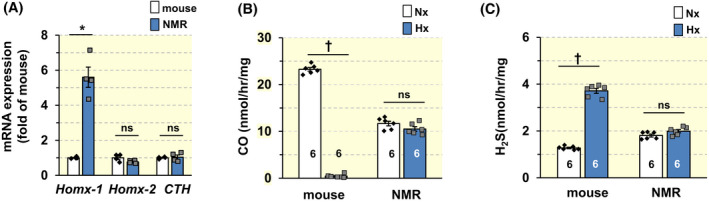
Analysis of mRNAs encoding CO and H_2_S producing enzymes and effects of hypoxia on CO and H_2_S in NMRs and BL6 mice. (A) *hmox‐1*, *hmox‐2*, and *CTH* mRNAs encoding HO‐1, HO‐2, and cystathionine ϒ‐lyase in CBs of BL6 and NMRs. Data were normalized to *18S* mRNA and presented as fold change of BL6 CBs. *N* = 4 animals in each group. (B, C) Effects of hypoxia (PO_2_ ~ 40 mmHg) on CO (B) and H_2_S (C) abundances in BL6 and NMR liver tissues. *N* = 6 animals each; ^†^ *p* < 0.01; *p < 0.05; n.s., *p* > 0.05, Mann–Whitney test in A, and two‐way ANOVA followed by Holm‐Sidak test in B and C. In B, for factor of species, *F*_(1,20)_ = 3.484, *p* = 0.077; for treatment, *F*_(1,20)_ = 883.005, *p* < 0.001; for species × treatment, *F*_(1,20)_ = 720.999, *p* < 0.001. In C, for factor of species, *F*_(1,20)_ = 69.55, *p* < 0.001; for treatment, *F*_(1,20)_ = 336.25, *p* < 0.001; for species × treatment, *F*_(1,20)_ = 247.2, *p* < 0.001.

Emerging evidence suggests that the regulation of CB sensitivity to hypoxia involves carbon monoxide (CO) and H_2_S gas messenger signaling pathways.^23^ Therefore, we next examined whether altered CO‐H_2_S signaling contributes to the blunted CB response to hypoxia in NMRs. Endogenous CO is produced by hemeoxygenase (HO)‐1 and HO‐2, and CSE is a major H_2_S producing enzyme in the CB.^23^ Therefore, *Hmox1*, *Hmox2*, *CTH* mRNAs encoding HO‐1, 2, and CSE, respectively, were determined in CBs of NMRs and mice. Transcript abundance was normalized to *18S* mRNA. *Hmox1*, which encodes HO‐1, was ~6‐fold higher in NMR than mouse CBs, whereas *Hmox‐2* and *CTH* abundances were comparable between CBs of both species (Figure 6A; *p* < 0.05; *n* = 4 animals for each species). Technical difficulties with antibodies precluded the analysis of HO‐1, HO‐2, and CSE proteins in NMR CBs.

CO inhibits CSE and reduces H_2_S in the CB.^23^, ^24^ Hypoxia inactivates HO‐2 and reduces CO production thereby releasing inhibition on CSE. This leads to increased H_2_S production, which in turn stimulates CSN activity.^24^ On the other hand, hypoxia has no effect on CO produced from HO‐1, because it lacks O_2_‐sensitive heme regulatory motifs (HRM).^25^ Because NMR CBs have elevated *Hmox‐1*, which encodes O_2_ insensitive HO‐1, we hypothesized that hypoxia would not effectively alter CO levels in NMRs. Testing this possibility using biochemical assays requires pooling of several CB tissues from numerous animals, which was not possible in our study due to the limited availability of NMRs. We have previously used rat pheochromocytoma (PC)‐12 cells as a substitute for CB glomus cells.^26^ However, we are not aware that PC12 cells express HO isoforms and CSE. Interestingly, liver tissue expresses a high abundance of HO‐2^27^ and CSE.^28^ In CBs, hypoxia reduces CO production by directly inhibiting HO‐2 and increases H_2_S levels^24^ and in liver homogenates in response to chronic intermittent hypoxia through ROS mechanisms.^29^ Together, these findings indicate similar interactions between CO and H_2_S in liver as in the CB. Therefore, we utilized liver tissues from NMRs and mice to assess the effects of hypoxia on CO and H_2_S signaling. In this analysis, we found that hypoxia reduced CO and increased H_2_S in mice but not in NMR liver (Figure 6B,C; *p* < 0.01; *n* = 6 NMRs and mice each).

### 2.6 An HO inhibitor improves CB response to hypoxia and HVR in NMRs

**FIGURE 7:**
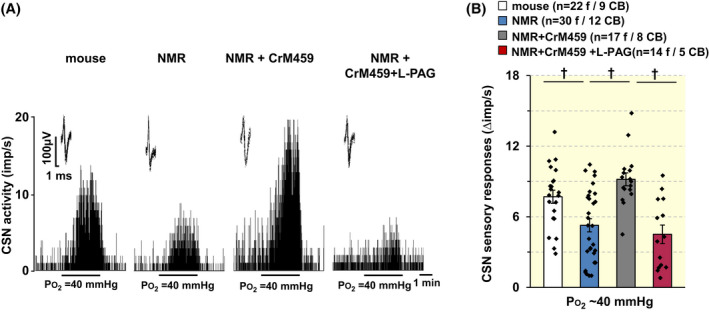
An HO‐inhibitor improves CB response to hypoxia in NMRs. (A). Examples of carotid body sensory nerve (CSN) response to hypoxia (medium PO_2_ ~ 40 mmHg) in NMRs treated with chromium (III) mesoporphyrin IX chloride (CrM459), a pan HO inhibitor either alone or combined with l‐propargylglycine (l‐PAG), an inhibitor of cystathionine γ‐lyase (CSE). CrM459 (1 mg/kg) and l‐PAG (30 mg/kg) were given IP (intraperitoneal) to urethane anesthetized NMRs 1 h before harvesting CBs. Integrated CSN action potential frequency presented as impulses per second (imp/s). *Insets*: superimposed action potentials of a “single” fiber from which data were derived. (B). Individual and average data (mean ± SEM) of CSN responses to hypoxia. Data represent hypoxia‐evoked CSN response minus baseline CSN activity (Δ impulse/s). Numbers in parenthesis represent the number of fibers and number of CBs from 14 NMRs and 5 BL6 mice. ^†^ *p* < 0.01, one‐way ANOVA followed by Holm‐Sidak test.

We next assessed whether increasing H_2_S with an HO inhibitor improves CB response to hypoxia in NMRs. To test this, NMRs were treated with chromium (III) mesoporphyrin IX chloride (CrM459; 5 mg/kg; i.p), a pan HO inhibitor,^23^, ^30^, ^31^ either alone or in combination with l‐propargyl glycine (l‐PAG; 30 mg/kg; I.P.), an inhibitor of H_2_S synthesis from CSE.^23^, ^32^ CBs were harvested 1 h after administration of these compounds and CSN responses to hypoxia were determined. The CSN response to hypoxia was markedly improved in HO inhibitor‐treated NMRs compared to vehicle‐treated controls, and this effect was blocked with l‐PAG (Figure 7A,B; *p* < 0.01; see Figure 7 for number of animals in each group).

**FIGURE 8:**
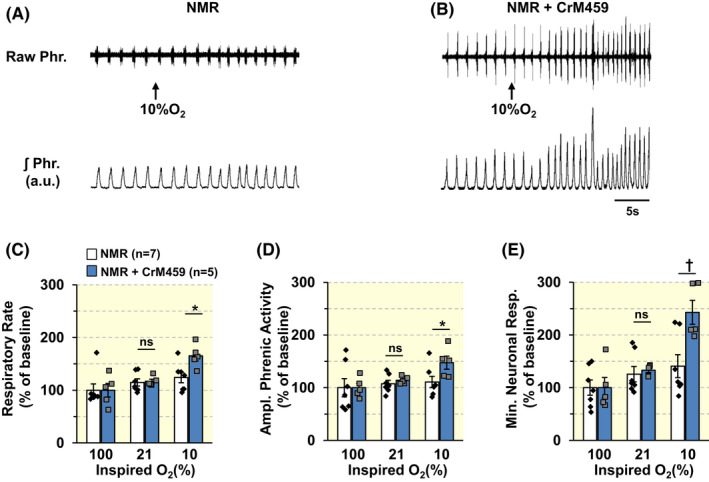
An HO inhibitor improves phrenic nerve responses to hypoxia in NMRs. (A, B). Examples of efferent phrenic nerve responses to 10% inspired O_2_ in urethane anesthetized, spontaneously breathing in NMRs treated with either vehicle (A) or CrM459, an HO‐inhibitor (B). Raw Phr., phrenic nerve action potentials. ʃPhr., integrated efferent phrenic nerve activity (arbitrary units a.u.). Arrows indicate application of 10% O_2_ gas mixture. (C–E). Individual and average data (mean ± SEM) of changes in respiratory rate (RR; phrenic burst frequency/min; (C), tidal amplitude of the phrenic nerve activity (D), and minute neural respiration (E) of NMRs treated with either vehicle (*n* = 5) or CrM459(*n* = 7) in response to a range of inspired O_2_ levels. Data presented as percent of phrenic nerve activity during breathing 100% O_2_. ^†^ *p* < 0.01; *p < 0.05; n.s., *p* > 0.05, two‐way ANOVA with repeated measures followed by Holm‐Sidak test. In B, for factor of treatment, *F*_(1,20)_ = 1.417, *p* = 0.261; for oxygen level, *F*_(2,20)_ = 13.12, *p* < 0.001; for treatment × oxygen level, *F*_(2,20)_ = 3.434, *p* = 0.05. In C, for factor of treatment, *F*_(1,20)_ = 5.374, *p* = 0.043; for oxygen level, *F*_(2,20)_ = 2.732, *p* = 0.089; for treatment × oxygen level, *F*_(2,20)_ = 0.751, *p* = 0.485. In D, for factor of treatment, *F*_(1,20)_ = 5.267, *p* = 0.045; for oxygen level, *F*_(2,20)_ = 15.339, *p* < 0.001; for treatment × oxygen level, *F*_(2,20)_ = 5.644, *p* = 0.011.

Consistent with this improved CB response to hypoxia, HO inhibitor enhanced the HVR in NMRs (Figure 8A,B; absolute values of phrenic nerve activity in Table 5). The improved HVR was due to increased respiratory rate (i.e., phrenic burst frequency) and tidal phrenic amplitude (Figure 8C–E and Table 5).

**TABLE 5:** Phrenic nerve responses to three levels of inspired O_2_ in urethane‐anesthetized NMRs treated with either vehicle or with CrM459, a HO inhibitor

	100% O_2_	21% O_2_	10% O_2_
*Vehicle (n = 7)*			
RR (breaths/min)	48 ± 5.7	56 ± 10.0	62 ± 13.2
Tidal Ampl (a.u.)	2.9 ± 0.5	3.0 ± 0.4	3.0 ± 0.4
MNR (a.u.∙min)	133 ± 19.2	159 ± 23.3	177 ± 29.8
*CrM459 (n = 5)*			
RR (breaths/min)	55 ± 3.9*	64 ± 3.1	90 ± 4.7
Tidal Ampl (a.u.)	3.8 ± 0.3^ns^	4.3 ± 0.5	5.5 ± 0.5
MNR (a.u.∙min)	210 ± 27.3*	279 ± 38.6	493 ± 45.7

*Note*: Numbers in parenthesis represent the number of NMRs.

Abbreviations: MNR, minute neuronal respiration; RR, respiratory rate; Tidal Ampl, tidal phrenic amplitude (arbitrary units; a.u.).

* *p* < 0.05.

^ns^ *p* > 0.05, vehicle vs. HO‐inhibitor Mann–Whitney test.

### 2.7 Carbonic anhydrase is involved in augmented CB response to CO_2_ in NMRs

**FIGURE 9:**
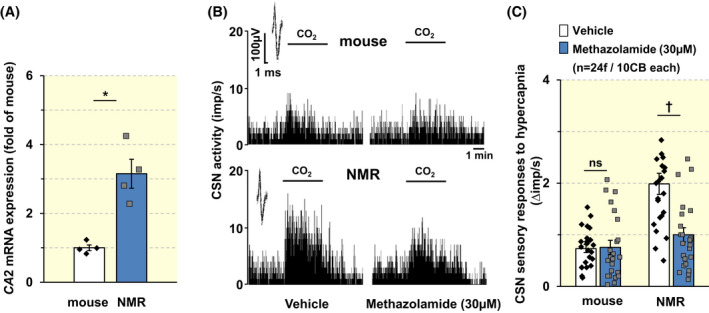
mRNA analysis of *CA‐2* in the carotid bodies (CBs) and the effect of methazolamide on CB sensory nerve (CSN) response to CO_2_ in NMRs. (A) *CA*2 mRNA abundance was determined by quantitative RT‐PCR assay in the CBs of BL6 mice and NMRs (*n* = 4 BL6 mice and 4NMRs). Data were normalized to *18S* mRNA and expressed as fold change of BL6 CBs; *p < 0.05, Mann–Whitney test. (B). Examples of CSN response to hypercapnia (medium PCO_2_ ~ 65 mmHg) in the presence of either vehicle (saline, *left*) or methazolamide, a CA inhibitor (*right*) in a BL6 mice (*upper panel*) and an NMR (*lower panel*). Integrated CSN action potential frequency is presented as impulses per second (imp/s). *Insets*: superimposed action potentials of a “single” fiber from which data were derived. (C). Individual and average data (mean ± SEM) of CSN response to hypercapnia (medium PCO_2_ ~ 65 mmHg) in the presence of either vehicle or methazolamide. Numbers in parenthesis represent the number of fibers and number of CBs from *n* = 5 BL6 mice and NMR each); ^†^ *p* < 0.01; n.s., *p* > 0.05; two‐way ANOVA with repeated measures followed by Holm‐Sidak test. For factor of species, *F*_(1,46)_ = 18.109, *p* < 0.001; for treatment, *F*_(1,46)_ = 19.942, *p* < 0.001; for species × treatment, *F*_(1,46)_ = 19.379, *p* < 0.001.

Carbonic anhydrases (CAs) are zinc‐containing enzymes that catalyze the conversion of CO_2_ to bicarbonate and hydrogen ions.^33^, ^34^ The CB response to CO_2_ involves CA.^35^, ^36^ We next examined whether CA contributes to the augmented CB response to CO_2_ in NMRs. mRNA encoding *CA2* was analyzed in CBs from NMRs and mice. *CA2* abundance was higher in NMR compared to mice CBs (Figure 9A; *p* < 0.05; *n* = 4 each species). Non‐specific staining with commercially available anti‐CA‐2 antibodies precluded analysis of CA‐2 protein in NMR CBs. However, methazolamide (30 μM), a membrane‐permeable CA inhibitor, reduced the augmented CSN response to hypercapnia in NMRs but not in mice compared to vehicle‐treated controls (Figure 9B,C; Vehicle vs. methazolamide NMRs *p* < 0.01; mice *p* > 0.05; *n* = 5 for mice and 6 for NMRs).

## 3 DISCUSSION

Previous studies have reported that NMRs manifest a blunted HVR and HCVR^3^, ^37^, ^38^; however, the mechanisms underlying this phenomenon are largely unknown. The current study addressed this knowledge gap by testing the hypothesis that attenuated hypoxic and CO_2_ sensing by the CB chemoreceptor contributes, in part, to the blunted HVR and HCVR in NMRs. Our study had several salient findings. First, and consistent with our hypothesis, the CB response to hypoxia was attenuated in NMRs compared to mice, indicating that the blunted HVR in NMRs is partially due to reduced CB sensitivity to hypoxemia. We further found that the mechanism underlying this blunted response involves HO signaling because a pan‐HO‐inhibitor improved CB sensitivity to hypoxia and HVR became detectable in NMRs. On the other hand, HCVR and CB response to CO_2_ was augmented in NMRs compared to mice, which may be due to altered CA signaling.

### 3.1 The CB response to hypoxia

The following findings demonstrate that NMRs exhibit impaired CB responses to hypoxia: first, NMR ventilation was less inhibited by brief hyperoxia than in mice (Dejour's test), which is an indirect measure of CB sensitivity to O_2_. Second, the CSN response to graded hypoxia was attenuated in NMRs. Consistent with earlier reports,^5^, ^7^ we also found a near absence of HVR in NMRs. This blunted HVR may be partially due to reduced body temperature since conscious NMRs cease thermoregulation and reduce their body temperature to near ambient temperatures in hypoxia.^39^ However, our study suggests that this is unlikely because we determined the HVR in anesthetized animals, while maintaining body temperature at 33°C in NMRs and 38°C in mice, which are their respective body temperatures.^21^ On the other hand, the blunted HVR is likely not due to anesthesia because previous studies reported similar impairment of the HVR in un‐anesthetized NMRs.^3^, ^37^, ^38^ Given that the CB chemoreflex is a major driver of the HVR,^14^, ^40^ it is therefore likely that the attenuated hypoxic sensitivity of the CB in part contributes to the near absence of HVR in NMRs. Whether NMRs also have impaired processing of CB sensory nerve information in the central nervous system remains to be investigated.

Being burrow‐dwelling animals, hypoxic lifestyle of NMRs may have impacted the morphological phenotype of their CBs. Indeed, prolonged exposure to hypoxia increases CB size in animals^41^ as well as in humans.^42^ Consistent with this, CBs were bigger, and the number of glomus cells was higher in NMRs compared to mice (Figure 5). Interestingly, NMR glomus cells displayed a dispersed pattern, as opposed to the clustered pattern in mice CBs, which is typical of most adult mammalian CBs.^14^ Glomus cells are of secretory phenotype,^14^ and are connected via gap junctions, allowing electrical coupling at rest. Hypoxia uncouples glomus cells and increase coupling resistance.^43^ The dispersed pattern of glomus cells in NMRs might reflect the impact of hypoxia resulting from life in crowded underground burrows. Interestingly, although the number of glomus cells is higher in NMR CBs, the ratio of TH to the CB volume was similar in NMR and mice, suggesting that the blunted hypoxic sensitivity of NMR CBs is likely due to defective hypoxia sensing at glomus cells (see below) and not fewer chemosensitive cells in NMRs per se.

### 3.2 Cellular mechanisms underlying the blunted CB response to hypoxia in NMRs

Recent studies suggest that CB sensory nerve excitation by hypoxia involves O_2_‐dependent interactions between CO and H_2_S in glomus cells.^23^, ^24^, ^44^, ^45^, ^46^ HO‐2 and CSE are the major enzymes that produce CO and H_2_S, respectively, in CB glomus cells.^23^, ^47^ While CO is a physiological inhibitor of the CSN response to hypoxia,^47^ H_2_S, like hypoxia, stimulates CSN activity in several mammalian species.^48^ Specifically, in normoxia, CO generated by HO‐2 inhibits H_2_S generation from CSE, thereby keeping the CSN activity low. On the other hand, hypoxia reduces CO production by inactivating HO‐2, thereby lifting inhibition on CSE, and the ensuing increase in H_2_S stimulates CSN activity.^24^

We found that, unlike in mice, hypoxia neither reduced CO production nor increased H_2_S in NMRs (Figure 6B,C). The absence of CB response to hypoxia in NMRS is likely due to the inhibitory action of CO produced by HO‐1, which is not inhibited by hypoxia.^25^ Indeed, *Hmox‐1* mRNA encoding HO‐1 was higher in NMR CBs than mice. Biochemical assays showed the absence of the effects of hypoxia on CO and H_2_S levels in NMR compared to mice. Notwithstanding the limitation of using liver tissue for biochemical assay, these results indicate O_2_‐insensitive CO generation from HO‐1 by inhibiting CSE reduces H_2_S production in NMR CB. The reduced H_2_S production might account for the blunted CSN excitation by hypoxia in NMRs. Such a possibility was supported by the finding that a pan HO‐inhibitor markedly improved CB hypoxic response in NMRs and l‐PAG, a CSE inhibitor blocked the effect of the HO inhibitor (Figure 7). The improved CB hypoxic response was associated with enhanced HVR in NMRs treated with HO inhibitor (Figure 8). It should be noted that *Hmox‐1* upregulation in NMR CB was not associated with increased baseline CO levels (Figure 6B), which might be due to incomplete translation of the *Hmox‐1* gene to HO‐1 protein, a possibility that requires further investigation.

### 3.3 The HCVR and CB responses to CO_2_ in NMRs

It is well established that burrowing rodents have relatively higher resting PaCO_2_ ^37^ levels and attenuated HCVRs.^49^, ^50^, ^51^ Interestingly, we found PaCO_2_ was not elevated in NMRs, which is consistent with a previous study (see Table 1 in Pamenter et al. 2019).^52^ Conversely, whereas two previous studies in whole animals have reported that NMRs have a blunted HCVR that does not manifest below 10% inhaled CO_2_,^6^, ^7^ whereas we report an augmented HVCR in NMRs. This discrepancy may be due to differences in the duration of hypercapnia exposure. For example, a previous study^6^ examined breathing responses in NMRs to 1 h of hypercapnia, whereas in the present study we tested the effects of 5 min of hypercapnia on ventilation (i.e., on phrenic nerve activity). In addition, this previous study^6^ used awake animals whereas our preparation was anesthetized. The other earlier study^5^ also employed anesthetized animals but only measured breathing empirically via remote observation, which prevented this investigation from reporting tidal volume changes with hypercapnia. Thus, it is not possible to directly compare this study to our present findings.

In other species, acute hypercapnia increases ventilation for a few minutes but prolonged hypercapnia depresses ventilation.^53^ It is likely the depressed HCVR reported in an earlier study^6^ in awake NMRs is due to prolonged hypercapnic challenge (1 h). NMRs likely experience hypoxia and hypercapnia simultaneously in their natural burrowing environment. The augmented HCVR likely increases O_2_ delivery to the lungs through a left‐shifted O_2_‐Hb dissociation curve,^54^ thereby reducing the impact of hypoxia. The enhanced HCVR may be an important contributing factor to the hypoxia tolerance of NMRs.^3^, ^4^, ^5^

Hypercarbia (i.e., elevated arterial blood CO_2_) stimulates CSN activity, albeit to a lesser extent than hypoxemia in most adult mammals.^14^ We report that, unlike hypoxia, the CB response to CO_2_ is augmented in NMRs compared to mice. This is important because it demonstrates that NMR CBs exhibit selective impairment of their ability to detect hypoxia, but not CO_2_. Unlike the response to hypoxia, the CB response to CO_2_ is relatively underexplored.^14^ However, the available information suggests CB activation by CO_2_ involves CA activity.^36^, ^55^ Intriguingly, NMR CBs have higher *CA‐2* mRNA abundance than mice, suggesting divergence in the function of this pathway between species. Unfortunately, technical problems with antibodies precluded the analysis of CA2 protein in CBs; however, methazolamide, a membrane‐permeable CA inhibitor reduced the enhanced CB response to CO_2_ in NMR CB preparations. Therefore, we propose that elevated CA‐2 leads to greater hydration of CO_2_, resulting in an accumulation of H^+^. H^+^ in turn depolarize glomus cells by inhibiting either TASK‐like K^+^ channels^56^ or acid‐sensing ion channels (ASICs),^57^ and thereby contribute to the augmented CO_2_ response of the NMR CB. However, further studies are needed to test this possibility by evaluating whether NMR glomus cells express TASK or ASIC channels and whether they contribute to the augmented CB CO_2_ response.

In summary, our results support the hypothesis that the blunted HVR in NMRs is associated with attenuated CB sensitivity to hypoxia. On the other hand, and contrary to our hypothesis, the HCVR and CB CO_2_ sensitivity are augmented in NMRs. As a largely subterranean species, NMRs likely experience prolonged periodic hypoxia and hypercapnia in their natural burrows. Although much is known about the impact of intermittent hypoxia, such as that experienced with obstructive sleep apnea on CB function and the HVR,^58^, ^59^, ^60^ little is known regarding the physiological consequences of long‐term exposures to a combination of periodic hypoxia and hypercapnia such as that experienced in burrowing animals. NMRs are relatively resistant to aging, neurodegeneration, and devastating diseases such as cancer,^61^ all of which have been linked to derangements in cellular oxygen handling. Future studies on experimental animals treated with long‐term periodic hypoxia and hypercapnia simulating the burrowing environment may thus provide mechanistic insights on how and why NMR are less susceptible to such diseases and pathologies.

## 4 METHODS

### 4.1 Preparation of animals

Experimental protocols were approved by the Institutional Animal Care and Use Committee of the University of Chicago (Protocol # ACUP 71811, approved on February 27, 2019). Experiments were performed on adult male NMRs (males; body weight 45.2 ± 1.6 g, and 1–3 years old; bred at the University of Ottawa, and reared at the University of Chicago, Animal Resource Center). BL6 mice (males; 28.4 ± 0.8 g, 4–6 months old, purchased from Charles River Laboratories, USA).

### 4.2 Measurements of phrenic nerve activity

Animals were anesthetized with intraperitoneal injections of urethane (1.2 g/kg). Supplemental doses (10% of the initial dose of anesthetic) were given when corneal reflexes or responses to toe pinches were observed. Animals were placed on a warm surgical board. The trachea was cannulated, and animals were allowed to breathe spontaneously. Core body temperature was monitored by a rectal thermistor probe and maintained at 38°C (mice) or 33°C (NMR) by a heating pad (RightTemp, Kent Scientific, Torrington, CT). The phrenic nerve was isolated unilaterally at the level of the C3 and C4 spinal segments, cut distally, and placed on bipolar stainless‐steel electrodes. Integrated efferent phrenic nerve activity was monitored as an index of respiratory neuronal output. The electrical activity was filtered (band pass, 30 Hz −10 kHz), amplified (P511K, Grass Instrument, West Warwick, RI), and passed through Paynter filters (time constant of 100 ms; CWE Inc.) to obtain a moving average signal. Data were collected and stored in a computer for further analysis (PowerLab/8P, AD Instruments Pty Ltd, Australia). Phrenic nerve activity (burst frequency, an index of respiratory rate, bursts/min); tidal phrenic nerve activity (arbitrary units, a.u.); and minute neuronal respiration (MNR = RR × tidal phrenic nerve activity) were analyzed. The effects of different O_2_ levels (21% or 10% O_2_‐balanced N_2_), and hypercapnia (3%, 5%, or 10% CO_2_‐balanced O_2_) on phrenic nerve activity were determined. Gases were administered through a needle placed in the tracheal cannula and gas flow was controlled by a flow meter. To examine the responses to different O_2_ levels, baseline phrenic nerve activity was monitored while animals breathed 100% O_2_ for 3 min. Subsequently, inspired gas was switched to 21% or 10% O_2_ for 3 min. The duration of 3 min for hypoxia was chosen because a longer duration of hypoxic exposure (>5 min) in anesthetized mice leads to hypotension which confounds the interpretation of results. For hypercapnic responses, 5 min of 3%, 5%, or 10% CO_2_‐balanced O_2_ was preceded with exposure to 100% O_2_ for 3 min. At the end of the experiment, animals were killed by overdose of urethane (>3.6 g/kg, i.p.).

### 4.3 Measurements of arterial blood gases

Arterial blood (0.1 ml) was collected in the anesthetized animals at the end of 3 or 5 min of gas challenges via a catheter (PE‐10) inserted into the femoral artery. Blood gases (PaO_2_, PaCO_2_, and pH) were determined by a blood gas analyzer (ABL‐80, Radiometer, Copenhagen, Denmark). Blood gas analyzer provides data corrected at 37°C as well as normal body temperature of the animals. Two to four blood samples were collected in each experiment.

### 4.4 Recording of CB sensory nerve (CSN) activity

The CSN activity was recorded from CBs ex vivo as described previously.^62^ Briefly, CBs (two CBs from a given animal) along with the sinus nerves were harvested from anesthetized animals, placed in a recording chamber (volume, 250 μl), and superfused with warm bicarbonate‐based physiological saline (35°C) at a rate of 3 ml/min. The composition of the medium was (in mM): NaCl, 125; KCl, 5; CaCl_2_, 1.8; MgSO_4_, 2; NaH_2_PO_4_, 1.2; NaHCO_3_, 25; d‐glucose, 10; Sucrose, 5. The solution was bubbled with 21% O_2_/5% CO_2_. Hypoxic challenges were achieved by switching the perfusate to physiological saline equilibrated with the desired levels of O_2_. Oxygen levels in the medium were continuously monitored by a platinum electrode placed next to the CB using a polarographic amplifier (Model 1900, A‐M Systems, Sequim, WA). To facilitate the recording of clearly identifiable action potentials, the sinus nerve was treated with 0.1% collagenase for 5 min. Action potentials (1–3 active units) were recorded from one of the nerve bundles with a suction electrode and stored in a computer via a data acquisition system (PowerLab/8P). “Single” units were sorted based on the shape, height, and duration of the individual action potentials using the spike discrimination module. To examine CB responses to graded hypercapnia, baseline CSN activity was recorded while irrigating the CBs with a 100% O_2_ equilibrated medium for 3 min, followed by medium equilibrated with either 95% O_2_ + 5% CO_2_ or 90% O_2_ + 10% CO_2_ for 5 min. The PO_2_, Pco_2_, and pH of the medium were determined by a blood gas analyzer (ABL‐80).

### 4.5 CB morphology

CBs were harvested from anesthetized animals (urethane 1.2 g/kg, i.p.) perfused with heparinized saline followed by 4% paraformaldehyde. The protocols for fixation of CBs were essentially the same as described previously.^23^ Specimens were frozen in Tissue Tek (OCT; VWR Scientific), sectioned at 8 μm, and mounted on collagen‐coated coverslips. Mouse CB usually yields 7–8 sections. However, 4–5 sections representing the middle of each CB were chosen for morphometric analysis (4–5 sections per CB; 2CBs from each animal; *n* = 4 animals for each species). Sections were blocked in PBS containing 1% normal goat serum and 0.2% Triton X‐100, and then incubated with anti‐chromogranin A (CGA, 1: 1000; AB Cam) or with anti‐tyrosine hydroxylase (TH, 1:300; Sigma) antibody in PBS with 1% NGS and 0.2% Triton X‐100 at 4°C for 16 h. After washing in PBS, sections were incubated with Texas red‐conjugated goat anti‐rabbit IgG and FITC‐conjugated goat anti‐mouse IgG (1:250; Molecular Probes) in PBS with 1% NGS and 0.2% Triton X‐100 at room temperature for 1 h. After washing with PBS, sections were mounted in DAPI‐containing media and visualized using a fluorescent microscope (Eclipse E600; Nikon). Carotid body morphology, glomic volume, and glomus cell numbers were analyzed in adjacent sections using ImageJ (NIH) as described previously.^63^ Briefly, serial sections (minimum 4–5 sections from each CB) were imaged individually. For each image, the CB area was measured manually by tracing the periphery of the carotid body and glomic cell area was calculated by tracing the periphery of glomus cells stained with TH or CGA (marker proteins). CB and glomic cell volumes were calculated by sum of each area multiplied by thickness and number of sections as described.^63^

### 4.6 Measurements of mRNAs

Real‐time RT‐PCR was performed using a MiniOpticon system (Bio‐Rad Laboratories) with SYBR GreenER two‐step qRT‐PCR kit (#11764–100, Invitrogen). Briefly, RNA was extracted from CBs using TRIZOL and was reverse transcribed using superscript III reverse transcriptase. Primer sequences for real‐time RT‐PCR amplification were as follows: *CA2* forward: CAC CAA GTT GGC GGG AGC CTA T; *CA2* reverse: TCT CCA TTG GCA ATG GGG AAG TCC; *CTH* forward: TGC TTG GAA AAA GCA GTG GC; *CTH* reverse: CCT CTA GCA ATT TGG TTT TGG A; *Hmox ‐1* forward: GGA GCA GGA CAT GGC CTT CT; *Hmox ‐1* reverse: AGG TCA CCC AGG TAG CGG GT; *Hmox ‐2* forward: TGA AGG AAG GGA CCA AGG AAG; *Hmox ‐2* reverse: GTG GTC CTT GTT GCG GTC C; *18s* forward: CGC CGC TAG AGG TGA AAT TC; *18s* reverse: CGA ACC TCC GAC TTT CGT TCT. Relative mRNA quantification was calculated using the comparative threshold (CT) method using the formula “2−ΔΔCT” where ΔΔCT is the difference between the threshold cycle of the given target cDNA between BL6 and NMRs. The CT value was taken as a fractional cycle number at which the emitted fluorescence of the sample passes a fixed threshold above the baseline. Values were compared with an internal standard gene 18S. Purity and specificity of all products were confirmed by omitting the template and by performing a standard melting curve analysis.

### 4.7 Measurements of H_2_S production

H_2_S levels in the livers were determined as described previously.^23^, ^64^ Briefly, liver homogenates were prepared in 100 mM potassium phosphate buffer (pH 7.4). The enzyme reaction was carried out in sealed tubes flushed with either 100% N_2_ or 21% O_2_. The PO_2_ of the reaction medium was determined by a blood gas analyzer (ABL‐80). The assay mixture in a total volume of 500 μl contained (in final concentration) 800 μM l‐cysteine, 80 μM pyridoxal 5′‐phosphate, 100 mM potassium phosphate buffer (pH 7.4), and tissue homogenate (10 μg of liver protein). The reaction mixture was incubated at 37°C for 1 h and at the end of the reaction alkaline zinc acetate (1% wt/vol; 250 μl) and trichloroacetic acid (10% vol/vol) were added sequentially to trap H_2_S generated and to stop the reaction, respectively. The zinc sulfide formed was reacted sequentially with acidic N,N‐dimethyl‐*p*‐phenylenediamine sulfate (20 μM) and ferric chloride (30 μM) and the absorbance was measured at 670 nm using a microplate reader. A standard curve relating the concentration of Na_2_S and absorbance was used to calculate H_2_S concentration and expressed as nanomoles of H_2_S formed per hour per milligram protein.

### 4.8 Measurements of CO production

CO abundance was measured in the livers using a spectrophotometric procedure as previously described.^23^, ^64^ Reaction mixtures containing 10 μg liver protein, NADPH (1 mM), hemin (25 μM), and NADPH Regenerating System Solution (BD Biosciences) were equilibrated to 21% O_2_ or 100% N_2_ at 37°C in sealed tubes. CO generated in the reaction was trapped in a reaction mixture containing 25 μM leuco crystal violet, 200 μM palladate, and 4 μM iodate. CO concentrations were calculated from a standard curve relating CORM‐2 concentration to absorbance of 620 nm light.

### 4.9 Data analysis

In anesthetized animals, the following respiratory variables were analyzed: respiratory rate (phrenic bursts per minute), tidal amplitude of the integrated phrenic nerve activity (a.u., arbitrary units), and minute neural respiration (MNR, number of phrenic bursts per min, RR × tidal amplitude of the integrated phrenic nerve activity, a.u.). In a given animal, absolute values of phrenic variables the response to hypoxia or hypercapnia as well as normalized data as the percentage of the phrenic nerve activity while breathing 100% O_2_ were analyzed. CSN activity (discharge from “single” units) was averaged during 3 min of baseline and during the entire 3 min of hypoxic or 5 min of hypercapnic challenge and expressed as impulses per second. Each data point represents the average of two trials in each animal for a given gas challenge. Average data are presented as mean ± SEM. Statistical significance was assessed by *t*‐test, or Mann–Whitney test, or One‐Way or Two‐Way ANOVA followed by a *posthoc* test, or Two‐Way ANOVA with repeated measures followed by a *post hoc* test using SigmaPlot (version 11). *p* Values < 0.05 were considered significant.

## CONFLICT OF INTEREST

The authors declare no competing interests.
